# PROF. PhD. LUIZ ROHDE: FORMER PRESIDENT OF THE BRAZILIAN COLLEGE OF DIGESTIVE SURGERY

**DOI:** 10.1590/0102-672020230024e1742

**Published:** 2023-06-30

**Affiliations:** 1 Universidade Federal do Rio Grande do Sul, Department of Digestive Surgery – Porto Alegre (RS), Brazil.

**Figure:**
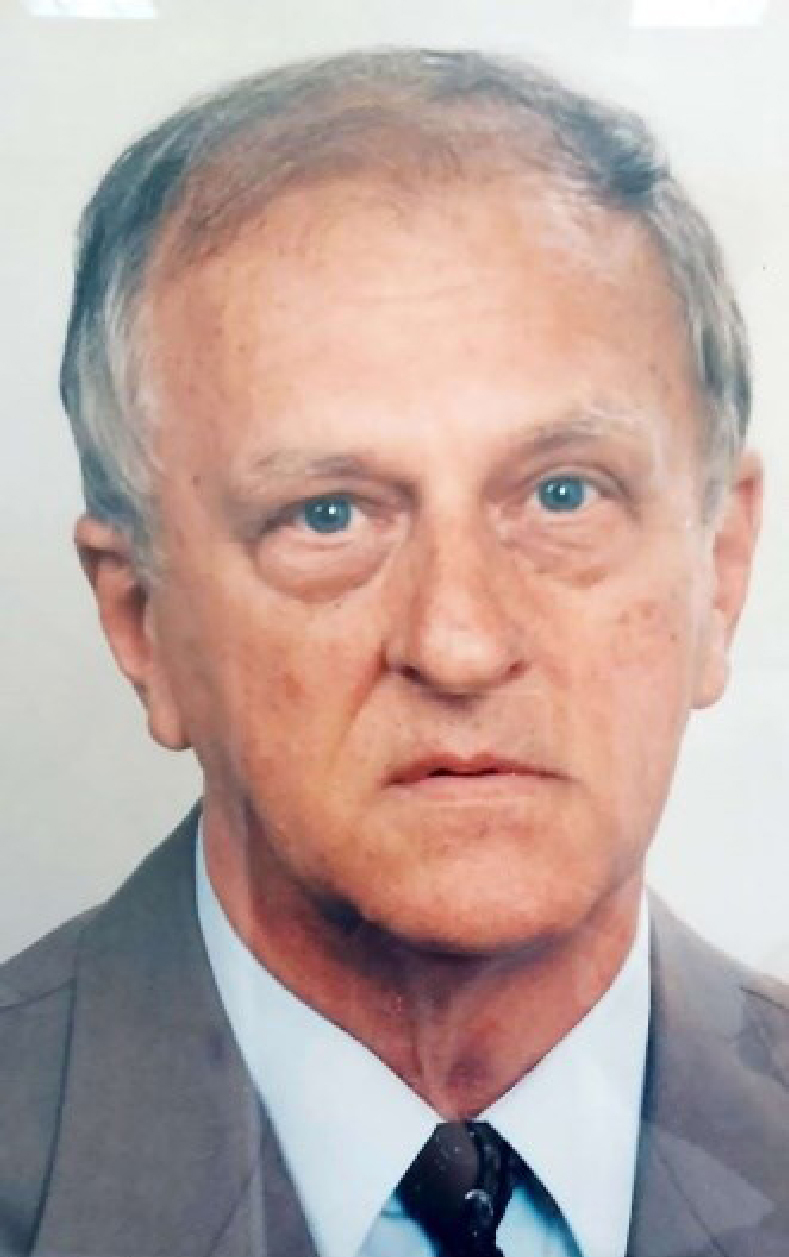


The German immigration to the state of Rio Grande do Sul (RS), Brazil, began in the 1920s. Between 1824 and 1922, 142 German colonies were created in Rio Grande do Sul, and it is estimated that, in this period, about 50 thousand Germans arrived in the state.

In this group of immigrants, Augusto Heinrich Rohde, Luiz Rohde's great-grandfather, arrived in 1857. On January 14, 1936, Luiz Rohde was born, the youngest son of Augusto Emílio and Erna Rohde, fruit of a union that generated eight children, in the locality of Paraíso do Sul, in the municipality of Cachoeira do Sul, 200 km from Porto Alegre (RS), in the central region of the state^1^.

After an initial school education in his region, in Cachoeira do Sul, Luiz completed the high school and soon the inclination to study medicine arose. Since then, it could be noted in the young Luiz Rohde marks that would be perennial in his character and in the example he would set for all those who were his students in the future: discipline, rectitude, education, and concern for the well-being of all with whom he had the opportunity to know and relate to.

He studied medicine at Universidade Federal de Santa Maria, where he joined in 1955 and concluded in 1961, having been chosen valedictorian of the class. At the same institution, another characteristic of Rohde was already evident: in the graduation speech, he drew attention to the need for creating a local university hospital to improve the training of doctors. Therefore, very early on he had the vision of what would be most important for the education of medical students. After completing the medical course, he was accepted, by a public tender at the University of São Paulo (USP), in the state of São Paulo (SP), in the residency in General Surgery, from 1962 to 1964.

It was also in 1964 that Luiz Rohde married Vera Guedes Paim, newly graduated from the dermatology residency at the Federal University of Rio Grande do Sul (UFRGS). They had two children: Luis Augusto and Luis Eduardo. They also chose to study medicine and pursue teaching like their father, and are currently full professors at UFRGS, respectively, in psychiatry and cardiology. The example of the medical parents stimulated the development of their careers, being nowadays two of the most outstanding professors at the university with worldwide relevance in their fields. In addition, they gave Luiz Rohde some of his greatest joys: his grandchildren Catarina and Pedro, and the latter is also studying medicine to take forward the third family generation in the profession chosen by Luiz Rohde.

In 1965, after returning to Porto Alegre, Rohde took over as an assistant professor at the Catholic College of Medicine of Porto Alegre (*Fundação Faculdade Católica de Medicina de Porto Alegre* – FFCMPA) (currently named Federal University of Health Sciences of Porto Alegre), working at this institution for 22 years.

There were hundreds of students and over a hundred residents in General and Digestive Surgery who had the privilege of being trained by Professor Luiz Rohde over the years. He was chosen as an honoree or class sponsor of the medical classes of 1967, 1968, 1969, 1972, 1973, 1974, 1980, 1981, 1983, and 1984. At FFCMPA, he developed his full potential as a teacher, surgeon, and researcher. He defended his Titular Professor thesis entitled *Contribuição ao estudo do tratamento cirúrgico dos tumores malignos da vesícula biliar* (“Contribution to the study of the surgical treatment of malignant tumors of the gallbladder”), in 1975.

While working at the Santa Casa de Misericórdia de Porto Alegre, a teaching hospital of that institution, he was head of the surgical wards number 30, 40 and 36, the latter known throughout the state since then as the “Infirmary of Professor Rohde,” where most of the graduates who opted for surgery longed to intern.

Rohde also dedicated himself to the Federal University of Rio Grande do Sul. Hired as a professor in 1978, by public tender, at a time when the General Hospital of Porto Alegre (*Hospital de Clínicas de Porto Alegre* – HCPA) was just beginning its activities, he worked in the infirmary of Santa Casa, under the coordination of UFRGS, thus being able to develop activities in both schools at the same hospital.

In 1986, he was elected head of the Department of Surgery of UFRGS, choosing to resign from FFCMPA and work exclusively at HCPA and UFRGS. A trajectory of great contributions of Luiz Rohde began there, and this university was the place of the greatest academic prominence of his teaching.

By a public tender, he became full professor of the Department of Surgery of UFRGS. In 1990, he coordinated the creation of the graduate program in Medicine: Surgery at the Master's level, being its first coordinator. In 1999, also due to his encouragement and effort, the graduate program began to offer the level of doctorate, and Rohde again coordinated the course, for another triennium.

During the period in which he was head of the Department of Surgery, he strongly acted on the teaching of General Surgery to students and residents, creating the subareas and groups of surgical activity in the digestive tract: Esophageal and Stomach Surgery Group, Liver Group, Pancreas and Bile Duct Group^2,3^ (being its first coordinator), and Bariatric Surgery Group. He has repeatedly advocated the importance of sub-specialization as a source of improvement in specialized medical care for patients and teaching and research in the Department of Surgery and in HCPA in the area of digestive surgery. Subsequently, he was elected head of the General Surgery Service of HCPA in 2005/2006.

In 1993, he was elected director of the School of Medicine (*Faculdade de Medicina* – FAMED) of UFRGS. Driven by the occasion of the centenary of UFRGS, in 1998, Rohde led a bold project to build the new FAMED building, bringing it to the HCPA, forming the current Health campus with other related schools. Thus, the visionary and pioneering side of Luiz Rohde appeared in a way that left an enduring legacy, gathering FAMED students in his teaching hospital in a definitive way.

Simultaneously, during his administration, the cornerstone for the construction of the Center of Experimental Medicine was laid, a building with six floors and which, after over 25 years, is currently a place where numerous clinical and experimental researches are undertaken in several areas, from surgery to endocrinology, genetics, among others. Certainly, it is a vital impulse in the scientific-academic production of the institution.

In 1988, he was one of the participants in the idealization and foundation of the Brazilian College of Digestive Surgery (CBCD), alongside his friend Henrique W. Pinotti and other colleagues, gathering prominent specialists in the field of Digestive Surgery from all over Brazil.

In 1994, he was elected president of the 1^st^ Brazilian Week of Digestive System, held in Porto Alegre, which was the seed that allowed us to be, in 2023, in the XXII Brazilian Week of Digestive System.

He was elected president of the Brazilian College of Digestive Surgery, term of office 1995 and 1996, and, following his predecessors, he greatly contributed to disseminating and valuing digestive surgery throughout the country.

Furthermore, he was a member of other national and international medical societies and an emeritus member of the Brazilian College of Surgeons, the Brazilian Medical Association, the Brazilian Society for the Development and Research in Surgery, the American College of Surgeons, the World Association of Hepato-Pancreato Biliary Surgery, and president of the Academy of Medicine of Rio Grande do Sul (2009-2010). He made numerous study trips inside and outside the country. In Brazil, he participated in congresses, courses, and medical journeys in most states, with about 350 lectures given, visiting its main medical and university centers, where he learned about issues related to medical care, scientific research, and medical education, and contributed to their solution.

He had almost a hundred scientific articles published in national and international journals. In 2005, he published the book *Rotina em Cirurgia Digestiva* (“Routine in Digestive Surgery”)^5^, which is already in its 5^th^ edition, being a reference for many students and residents in the field of Digestive Surgery.

In 2006, he founded the Digestive Surgery Service at HCPA, unanimously approved by the institution's specialties. Henceforth, over 50 residents have been trained in digestive tract surgery. During all these years, he was responsible for the surgical and academic training of numerous masters and doctors, some of them who are currently physicians and professors at FAMED and other educational institutions. In 2008, the renowned Council of UFRGS granted him the title of Professor Emeritus of the University.

In 2018, he was honored with the nomination of the Service, which, from then on, was called the Prof. Luiz Rohde Digestive Surgery Service.

We and so many others who know and relate to Professor Luiz Rohde have in him the example of leadership marked by education, rectitude of his conduct, and complete devotion to the art of operating and teaching. His time in the academy, in two Schools of Medicine, was enormously fruitful and continues to be up to now.

Better than talking more about Professor Luiz Rohde is to transcribe an excerpt from one of his many speeches^4^. Perhaps, with it, we can capture some of the essence of this master in the art of operating and teaching:

“I understand medical education as an integrated whole and not as a simple sum of contents of medical disciplines and specialties. In teaching, in graduate programs and in residency, practice is the great motivator of learning. Problem-based learning. The end result should be the surgeon with deep knowledge, technical competence, ethical humanitarian training, and socially responsible performance. Physicians, especially surgeons, when making their decisions based on evidence, use science; and when they apply these decisions, they put art into practice. Science and art applied with competence, dedication, respect, and compassion are the characteristics that the best surgeons should have.”

Teaching is done by words and example. Dear Master Rohde, your example lives on in all of us.
